# Endocrine-Disrupting Air Pollutants and Their Effects on the Hypothalamus-Pituitary-Gonadal Axis

**DOI:** 10.3390/ijms21239191

**Published:** 2020-12-02

**Authors:** Elizabeth C. Plunk, Sean M. Richards

**Affiliations:** 1 Department of Environmental Medicine, University of Rochester Medical School, Rochester, NY 14642, USA; 2 Department of Biological, Geological and Environmental Sciences, University of Tennessee-Chattanooga, Chattanooga, TN 37403, USA; seanrichards.utc@gmail.com; 3 Department of Obstetrics and Gynecology, College of Medicine, University of Tennessee Health Science Center, Chattanooga, TN 37403, USA

**Keywords:** endocrine disrupting chemicals, hypothalamus-pituitary-gonadal axis, air pollutants

## Abstract

Anthropogenic endocrine-disrupting chemicals (EDCs) can contaminate air, soil, and water. Human exposures to EDCs occur through inhalation, absorption, and ingestion. EDCs act by disrupting various pathways in the endocrine system. When the hypothalamic–pituitary–gonadal (HPG) axis is disrupted by EDCs, there can be effects on fertility in both men and women. Not only can fertility be indirectly affected by EDC disruptions of the HPG axis, but EDCs can also directly affect the menstrual cycle and sperm morphology. In this review, we will discuss the current findings on EDCs that can be inhaled. This review examines effects of exposure to prominent EDCs: brominated and organophosphate flame retardants, diesel exhaust, polycyclic aromatic hydrocarbons, cadmium and lead, TCDD, and polychlorinated biphenyls on fertility through alterations that disrupt the HPG axis and fertility through inhalation. Although the studies included herein include multiple exposure routes, all the studies indicate receptor interactions that can occur from inhalation and the associated effects of all compounds on the HPG axis and subsequent fertility.

## 1. Endocrine-Disrupting Chemicals

Endocrine-disrupting chemicals (EDCs) are exogenous chemicals that target the neuroendocrine and endocrine systems by disrupting endogenous hormone production, kinetics, signaling pathways [1] and (or) homeostatic systems [2]. Prominent exposure routes to EDCs include drinking contaminated water, breathing contaminated air, ingesting food, or contacting contaminated soil. People in occupations working with pesticides, fungicides, and industrial chemicals are at higher risk of exposure [3].

The endocrine system consists of a collection of glands that secrete hormones into the bloodstream which work as chemical messengers to regulate physiological functions throughout the body [4]. Similarly, the neurosecretory cells, glands, and non-endocrine cells of the neuroendocrine system secrete hormones into the bloodstream. Hormones work in a genomic and non-genomic manner. In the genomic mechanism, lipophilic hormones (e.g., steroid and thyroid hormones) bind directly to a receptor, which causes an alteration in gene expression [4,5]. In the non-genomic mechanism, gene expression is altered by intracellular signal transduction pathways (2nd messenger systems) when hydrophilic hormones (e.g., peptide or catecholamine) bind a receptor on the cell’s surface [4]. EDCs can alter the action of hormones in multiple ways. EDCs can affect hormone synthesis in the endocrine gland. Once the hormone is in the blood, EDCs can alter conjugation and binding to carrier proteins. At the target cell receptor, EDCs can compete with the endogenous hormone and cause antagonistic or agonistic target cell responses [6]. EDCs can also modify receptor concentrations on a cell or alter hormone metabolism [3,6]. In vitro and in vivo models can be used to demonstrate the complex pathways that EDCs affect, including (but not limited to) nuclear receptors estrogenic, anti-androgenic, thyroid, as well as pathways involving steroidogenic enzymes and neurotransmitters [3]. More specifically, EDCs can disrupt the actions of steroid hormones, which include estrogens and androgens [6], non-nuclear steroid hormone receptors, non-steroid receptors, and orphan receptors (receptors for which ligands have not been identified) [2]. 

Due to the regulatory roles of estrogens and androgens in reproduction, EDCs can have negative effects on reproductive health through anti-estrogenic, estrogenic, anti-androgenic and androgenic effects [6]. EDCs can disrupt the production and signaling of androgens and can disrupt the development of the male reproductive tract and external genitalia, leading to adverse effects on male reproduction and fertility [7]. Female subfertility or infertility can be disrupted by EDCs by causing altered hormone production, menstrual cycle abnormalities, anovulation, and early reproductive senescence [8]. The most vulnerable periods of exposure to EDCs are prenatally, by the transplacental transfer of EDCs, and postnatally via breast feeding [3,4,6,9]. Disturbance to hormonal activities during these times can lead to health problems including reproductive health and behavioral problems throughout life [3,4,6,9].

## 2. Hypothalamic—Pituitary—Gonadal Axis

The hypothalamic–pituitary–gonadal (HPG) axis, also referred to as the GnRH-gonadotropins, steroids axis [5], plays a critical role in reproduction in men and women. The main peptide hormones are gonadotropin-releasing hormone (GnRH), luteinizing hormone (LH), follicle-stimulating hormone (FSH), and the steroidal hormones are estrogen, progesterone, and testosterone, and proteins inhibins, activins, and follistatin [5,10]. 

The hypothalamus produces GnRH; the pituitary produces LH and FSH (i.e., gonadotropins); the gonads produce estrogens, progesterone, testosterone, and inhibins; and all tissues produce activins and follistatin [5]. The release of these hormones is regulated by feedback loops. Activins, inhibins, and follistatin hormones are responsible for paracrine regulation of spermatogenesis [5]. Activins stimulate the secretion of GnRH from the hypothalamus; follistatin has a high affinity for activins [11] and affects FSH secretion from pituitary cells [12]. The release of GnRH from the hypothalamus to the pituitary via the hypophyseal portal circulation stimulates the secretion of gonadotropins into the bloodstream when GnRH binds to the GnRH receptors (GnRHR) on the anterior pituitary [13], while LH and FSH are secreted in concert [14,15]. The bloodstream carries LH and FSH (bound to chaperone proteins) to the gonads, where they bind to the appropriate receptor and postnatally stimulate oogenesis or spermatogenesis [15]. Estrogens and testosterone along with inhibin are subsequently produced and comprise the negative feedback loop [14]. GnRH and gonadotropin are subsequently decreased by sex steroids and inhibin, respectively [16]. Indeed, sex steroids, activin, inhibin, and follistatin secretion are all necessary to maintain gonadotropin homeostasis through positive and negative feedback loops responsible for reproductive function [1].

The role of LH differs in males and females. In males, testicular cells include Leydig cells and Sertoli cells. Once LH has reached these cells, LH stimulates the production of testosterone in the testicular Leydig cells via 2nd messengers [5,6,13]. Sertoli cells are also targeted by LH, and they begin to produce androgen-binding protein once LH binds to its receptors [6,13]. The surface of Sertoli cells also has receptors for FSH, and once FSH has bound, it works simultaneously with the testosterone released from the Leydig cells to promote the proliferation of spermatogonia [13]. The release of testosterone creates a negative feedback loop by inhibiting the secretion of LH from the anterior pituitary [17]. In females, theca cells surround the ovarian follicle, and granulosa cells surround the oocyte. LH stimulates the production of androgens in theca cells of the primary follicles [15], and in the granulosa cells, LH stimulates the production of progesterone [13]. FSH stimulates the growth of ovarian follicles when binding to G protein-coupled receptors on granulosa cells, which stimulates the expression of aromatase enzymes [13]. The aromatase enzymes produced by the granulosa cells act on the androgens produced from the thecal cells and convert them to estradiol [13]. Clearly, the HPG axis is intricate and dependent on multiple pathways and feedback loops. Any perturbation of the axis will directly or indirectly affect fertility in men or women.

## 3. Fertility 

Alterations in concentrations of the hormones described above can lead to erroneous reproductive function and may result in infertility. Infertility is defined as unsuccessful fertilization after 12 months without using contraceptives; however, in women older than 35 the period of time changes to 6 months [18]. In couples of reproductive age, 10–15% are infertile due to female infertility, male infertility, or female and male infertility, making infertility nearly as common as diabetes mellitus [19]. In 2010 study, 6.0% of married women in the US between the ages of 15 and 44 were infertile, and 44% of women between the ages of 35–44 were infertile; meanwhile, 9.4% of men between the ages of 15–44 and 12% of men ages 25–44 were infertile [20]. In a 2015 study, 7% of males of the general population were reported as infertile [21]. Endocrine disorders, ovulatory disorders, endometriosis, pelvic adhesions, tubal blockage and abnormalities, and hyperprolactinemia are the most common causes of female infertility [22]. Although the most common symptoms of male infertility are poor sperm production, sperm motility, and sperm viability, the cause of male infertility is often referred to as idiopathic because mechanisms that cause the alterations in these sperm parameters are unknown [21].

## 4. EDCs in Air Pollution 

Anthropogenic EDCs are present in environmental pollutants from agriculture, industry, and consumer products [5] found indoors [23] and outdoors [24]. Air pollutants can exist as gases, liquid droplets, or solid particles [4], and are classified as gaseous pollutants, organic compounds, heavy metals, and particulate matter (PM) [25]. Food and water can become contaminated by air pollutants with common routes of exposure including ingestion and inhalation, leading to absorption through the gastrointestinal and respiratory tract, respectively [25].

More specifically, organic pollutant molecules in a gaseous phase can exist as volatile organic compounds (VOCs) or semi-volatile organic compounds (SVOCs) [4], and they can also attach to particulate matter (PM) such as dust, soil, acids, and some metals [26]. Domestic pesticide sprays, air fresheners, laundry detergents, household cleaners, paints, and plastics contain EDCs [4], and without adequate ventilation these consumer goods can heavily contribute to indoor air pollution [24].

## 5. Flame Retardants

Among the more potent estrogenic flame retardants are halogenated flame retardants (also known as organohalogen flame retardants), and organophosphorous flame retardants [27]. Brominated flame retardants (BFR) are able to bioaccumulate in the indoor environment due to their use in electronic equipment, textiles, and furniture; humans are exposed by inhalation of the contaminated air or particulate matter (PM) [28,29,30]. BFRs are lipophilic as well as persistent [31]. When products containing BFRs are no longer used they are typically recycled, incinerated, or placed in a landfill [27]. Controlled incineration decreases the release of toxic by-products into the ambient atmosphere whereas fugitive gases from poor-quality incineration release toxic by-products [27]. 

Polybrominated diphenyl ethers (PBDEs) are widely used BFRs with an estimated 209 congeners called brominated diphenyl ethers (BDE) [32,33,34] with weak estrogenic activities [33,35]. Degradation products of PBDEs are more toxic than the parent compound [27]. For example, Deca-PBDEs (10 bromines) degrade to Octa- and Penta-PBDEs (8 and 5 bromines, respectively), both of which are more toxic than the parent compound [36]. In addition, these congeners pose a different threat to reproductive health and have various half-lives in human serum, e.g., Deca-BDE (BDE209): 11–18 days; Octa-BDE (BDE196,197): over 68–120 days; Penta-BDE (BDE99,100): 2–26 years) [29]. Chronic exposure to PBDEs can occur indoors due to dust form [30] or by BFRs leaching into domestic environments [37]. In the non-industrialized country, Ghana, Asante et al. [38] found that mean levels of PBDEs in breast milk from 2004 to 2009 were 4.5 ng/g lipid weight (lw), while the range was 0.86–18 ng/g lw, and the levels measured significantly increased yearly. The studies described below are compiled and presented in Table 1 and Table 2.

### 5.1. Male-Specific Responses

PBDEs have been shown to uniquely affect men. Den Hond [29] collected semen, blood, and urine samples from 163 male patients recruited through four fertility clinics in Belgium. These men were unsuccessful in trying to impregnate their partner for 12 months and had suspected or documented decreased semen quality [29]. Along with biofluid samples, information on lifestyle, socio-economic status, health status, and living conditions were collected by questionnaires [29]. BDE209 and BDE 28 levels and testosterone levels were inversely related [29]. Furthermore, BDE209 levels were associated with a significantly greater risk of subfertility (*p* = 0.047) due to loss in sperm concentration and/or motility [29]. In contrast, serum levels of BDE153 and BDE154 showed a positive correlation with testosterone (*p* = 0.001, *p* = 0.002) and estradiol (*p* = 0.003, *p* = 0.04) [29]. 

Akutsu et al. [32] collected serum and sperm samples for 45 Japanese men ranging from 18 to 22 years old. TeBDE-47, PeBDE-99, PeBDE-100, and HxBDE-153 were the PBDE congeners monitored in the samples [32]. Sperm concentration (*p* = 0.002) and testis size (*p* = 0.01) were negatively correlated with BDE153 serum concentrations [32]. However, no relationship was found between the other congeners and sperm quality [32]. 

Hormone levels in 24 men of an infertile couple (male factor, female factor, or both) were compared with concentrations of BDE-47, 99, and 100 found in household vacuum bags [30]. FSH and LH serum concentrations were negatively correlated with concentrations of BDE-47 (*p* = 0.02, *p* = 0.004), 99 (*p* = 0.02, *p* = 0.002), and 100 (*p* = 0.01, *p* = 0.006) [30]. Similarly, Meijer et al. [39] studied how prenatal exposure to BDE-47, 99, 100,153, and 154 affected levels of testosterone, LH, and FSH in maternal serum and in boys at 3 and 18 months. In boys at 18 months of age, their testosterone levels were positively associated with the serum concentrations of BDE-154 in their mother’s serum [39].

Carignan et al. [40] analyzed urine samples for five metabolites of organophosphate flame retardants (PFRs) in 201 couples undergoing in vitro fertilization (IVF). High paternal urinary concentrations of one of the metabolites, bis(1,3-dichloro-2-propyl) phosphate (BDCIPP), correlated with a decline in the number of best-quality embryos [40]. However, there were no significant differences observed in successful implantation, clinical pregnancy, or live births [40]. 

To study the effects of BDE-99 exposure on male rats, Kuriyama and colleagues [34] treated rats by gavage on gestational day (GD) 6 with doses of 0.06 or 0.3 mg BDE-99/kg. On postnatal day (PND) 140, no effects were seen in testosterone (T) or LH levels in either group compared to controls [34].

### 5.2. Female-Specific Responses

Lefevre et al. [37] administered BFR dietary mixtures to female rats to represent the levels of BFR exposure from North American house dust. They were given a diet of 0, 0.06, 20, or 60 mg/kg/day BFR mixture from 2 to 3 weeks before mating until gestational day (GD) 20 [37]. A significant increase in the numbers of preantral and antral follicles (*p* = 0.038 and *p* = 0.024, respectively) as well as enlargements of the antral follicles was observed in BFR exposed groups [37]. In addition to ovarian changes, a significant increase in testosterone was also observed [37]. This study found that BFR exposure alters folliculogenesis and steroidogenesis in female rats [37]. 

2,2′,4,4′-tetrabromodiphenyl ether (PBDE-47) is an abundant PBDE congener found in human breast adipose tissue and maternal milk [41]. On gestation day 6 through PND 21, Talsness et al. [41] administered 140 or 700 µg/kg body weight (bw) PBDE-47 via drinking water to pregnant rats. On PND 38 the female offspring were sacrificed, and the ovaries were examined [41]. The group exposed to 140 µg/kg/bw PBDE-47 had a significant reduction in ovarian weight (*p* < 0.01) [41]. In the group exposed to 700 µg/kg/bw, a significant decrease in tertiary follicles (*p* < 0.05) and serum estradiol concentrations (*p* < 0.01) were observed [41]. No significant changes were observed in reproduction when exposed females were mated with non-exposed males [41]. 

Harley et al. [42] evaluated the relationship between fecundity in 223 pregnant Mexican-immigrant women living in California and levels of BDE congeners 47, 99, 100, and 153 in blood. No association was observed between BDE congener presence and menstrual cycle characteristics [42]. The increased levels of these congeners were positively associated with prolonged time to pregnancy [42]. 

Carignan et al. [43] collected urine samples from women undergoing in vitro fertilization (IVF) and quantified five PFR metabolites. Metabolite frequencies for bis(1,3-dichloro-2-propyl) phosphate (BDCIPP), diphenyl phosphate (DPHP), isopropylphenyl phenyl phosphate (ip-PPP) were significantly higher than tert-butylphenyl phenyl phosphate (tb-PPP), and bis(1-chloro-2-propyl) phosphate (BCIPP) [43]. An increase in the sum of metabolites and an increase in DPHP and ip-PPP individually had a negative association with successful IVF outcomes [43]. 

## 6. Diesel Exhaust and Polycyclic Aromatic Hydrocarbons

Diesel exhaust (DE) is a large source of outdoor air pollution containing polycyclic aromatic hydrocarbons (PAHs), sulfate, nitrate, heavy metals, ultrafine particles [44,45], and semi-volatile organic compounds (SVOCS) [46]. Diesel exhaust particles (DEPs) contain collections of carbon materials, ash, volatile organic compounds, and sulfur compounds [47]. The particles found in DE have estrogenic, anti-estrogenic, and anti-androgenic properties that affect gonadal steroidogenesis and gametogenesis [48,49,50]. Within the SVOCS themselves, *n*-alkanes, branched alkanes, alkyl-cycloalkanes, alkyl-benzenes, and PAHs are found [46]. While PAHs alone are EDCs, in this review, DE is first reviewed as a whole because the EDC effects of DE cannot be solely attributed to PAHs. The studies described below are compiled and presented in Table 1 and Table 2.

### 6.1. Male-Specific Responses

Exposure to 0.1 mg DEP/m3 diesel exhaust particles (DEPs) in utero from embryonic day 3 to 13 in male rats resulted in a decrease in daily sperm production due to the fewer number of Sertoli cells caused by alterations in gene expression [44]. Another study found that FSH serum levels and sperm production were decreased in male rats when exposed to total diesel exhaust (DE) as well as filtered exhaust which did not contain PM; however, testosterone and estradiol levels were increased compared to controls (*p* < 0.05) [51]. Exposure lasted three months beginning at birth, and they were exposed 6 h a day for 5 days a week via inhalation chambers [51]. LH was the only monitored hormone that significantly decreased (*p* < 0.05) in total DE exposed rats, suggesting inhibition of spermatogenesis [51]. Yoshida et al. [52] found, in male mice exposed to 0.3 mg DEP/m^3^ 12 h a day for up to 6 months, changes in Leydig cells, and after exposure to 1 mg DEP/m^3^, a reduction in LH receptor mRNA expression in Leydig cells [52].

Dzikowska et al. [53] exposed male rats to first generation diesel fuel containing 20% biocomponent (B20) via a whole-body inhalation chamber for 6 h a day for 5 days a week for 7 or 28 days. In the group exposed for 7 days, significantly higher concentrations of intratesticular and plasma testosterone and dihydrotestosterone were observed while no significant differences were observed in androgen receptor (AR) or estrogen receptors alpha and beta [53].

Li et al. [54] exposed male mice to clean air, low-dose (Low) nanoparticle-rich diesel exhaust (NR-DE), high-dose (High) NR-DE, and filtered (F) DE for 8 weeks. Mice exposed to High NR-DE had the highest levels of testosterone in comparison to controls and F-DE groups [54]. To examine this further, Li et al. [54] extracted interstitial cells from the males exposed to NR-DE, F-DE, or clean air, and these cells were incubated with or without human chorionic gonadotropin (hCG; 0.1 IU/mL) for 4 h. The hCG had no effect; High NR-DE exposed cells had increased testosterone production, and F-DE exposed cells had decreased testosterone production [54]. Furthermore, the expression of genes involved with testicular cholesterol synthesis; HMG-CoA, LDL-R., SR-B1, PBR, and P450scc, P450-17α, and 17β-HSD; were increased after High NR-DE exposure [54]. This suggests that as a result of the increase in testicular enzymes responsible for testosterone biosynthesis, NR-DE exposure increases testosterone biosynthesis [54]. 

Yoshida et al. [55] exposed murine Leydig TM3 cells from mouse testis to 10 µg/mL DEP for 24 h. A significant reduction in ERα mRNA expression was observed (*p* < 0.01) as well as a significant increase in P450 1A1 mRNA expression (*p* < 0.01) [55]. Meanwhile, to study the effects of DEP exposure in utero, Hemmingsen et al. [56] exposed pregnant mice to 20 mg/m^3^ diesel exhaust particles (DEPs) for one hour a day from GD 7–19. On PND 170 male offspring were sacrificed [56]. Prenatally exposed male mice had a decrease in daily sperm production; however, no significant differences were observed in plasma testosterone and estradiol concentrations [56]. Furthermore, no significant differences were seen in gene regulation of androgen receptor, anti-Mullerian hormone, estrogen receptor -α, estrogen receptor-β, FSH receptor, or LH receptor [56].

### 6.2. Female-Specific Responses

Tsukue et al. [57] exposed female mice to 0.3, 1.0, or 3.0 mg DEP/m^3^ for 12 h a day, 7 days a week for 4 months. After this exposure, one group of treated females were sacrificed and examined while the other females were mated with non-treated males [57]. Exposure group 1.0 mg DEP/m^3^ had significantly lighter uterine weights compared to the controls [57]. The offspring of the treated females and non-treated males were sacrificed on PND 30 or 70 [57]. The females from exposure group 3.0 mg DEP/ m^3^ sacrificed on PND 30 had significantly lower thymus and ovary weights [57]. Moreover, in groups exposed to 1.0 or 3.0 mg DEP/m^3^ vaginal opening occurred significantly earlier than in other exposure groups and controls indicating earlier sexual maturation [57].

Ogliari et al. [58] exposed groups of mice prenatally and postnatally to DE doses that correspond with the daily average PM_2.5_ levels humans are exposed to as reported by the World Health Organization (WHO). The number of primordial follicles was reduced (*p* </= 0.035) in groups of mice who were exposed to DE perinatally, postnatally, and both, and the proportion of primary follicles (*p* = 0.04) was diminished in mice exposed during pregnancy [58].

## 7. Polycyclic Aromatic Hydrocarbons

PAHs are organic compounds composed of two or more benzene rings and are arranged in various configurations [59]. Polycyclic aromatic hydrocarbons (PAHs) are air pollutants produced by the incomplete combustion of organic substances such as coal, oil, foods, tobacco, wood, as well as in vehicular emissions (including diesel exhaust) and petroleum-derived substances [60,61]. In 1976, the Environmental Protection Agency (EPA) published 16 PAH Priority Pollutants as representatives of PAHs that needed to be regulated [62]. 

PAHs are known endocrine disruptors, most commonly affecting estrogen receptors and aryl hydrocarbon receptors [63]. Reduced fertility has been shown in occupants of cities with heavy traffic related air pollution as well as if the resident lives closer to a major road [64,65]. In rodents, PAHs are known ovarian toxicants [60]. Tobacco smoke is also a source of PAH exposure; reproductive abnormalities in women are associated with smoking [60]. Benzo(a)pyrene (BaP) is a well-known PAH that can generate reactive oxygen species and create BP diol-epoxide-DNA adducts (BPDE) [66]. BaP exposure can come from automobile exhaust and cigarette smoke [67]. Human exposures to BaP range from 8.4 to 17 µg/person/day through the diet (Ramesh et al., 2011). A person who smokes a pack of cigarettes a day is assumed to add 0.1 µg exposure per day [68]. The studies described below are compiled and presented in Table 1 and Table 2.

### 7.1. Male-Specific Responses

Inyang et al. [69] exposed rats to 25, 75, and 100 μg BaP/m^3^ for 4 h a day for 10 days via inhalation. At 0, 24, 48, and 72 h after the end of exposure, blood samples were taken to measure testosterone and LH concentrations [69]. From hours 0 to 48 in exposures to 75 μg BaP/m^3^, male rats had decreased concentrations of plasma testosterone compared to controls [69]. Testosterone concentrations in male rats exposed to 75 μg BaP/m^3^ then increased by 72 h post-cessation of exposure, resulting in a higher mean concentration in the exposed group than in controls [69]. Plasma LH concentration had no significant increase at time 0, but as seen in testosterone, during time 24, 48, and 72 LH concentrations increased [69]. 

Han et al. [70] recruited 562 males who had unexplained infertility from hospitals affiliated with Nanjing Medical University. Urine samples were taken to measure four PAH metabolites: 1-naphthol (1-N), 2-naphthol (2-N), 2-hydroxyfluorene (2-OF), and 1-hydroxypyrene (1-OP) [70]. Blood samples were used to measure serum levels of FSH, LH, estradiol (E2), testosterone, and prolactin [70]. A significant positive correlation was observed between serum LH levels and urine concentrations of 1-OP (*p* = 0.048) [70]. 

Conti et al. [66] analyzed semen samples from 86 volunteers from Regalbuto (rural) and Melilli (industrial), Sicily. BaP Tetrol I-1 (TI-1) and BaP Tetrol II-2 (TII-2) are biomarkers of BaP exposure [66]. In Melilli men, the number of TI-1 adducts was greater, while in Regalbuto men the number of TII-2 adducts was greater. Furthermore, the sum of TI-1 and TII-2 adducts was significantly higher (confidence interval = 95%) in men from Regalbuto [66]. Conti et al. [66] found an inverse relationship between sperm motility and both TI1-1 and TII-2 adducts indicating exposure to BaP may negatively affect male fertility.

Radwan et al. [71] collected urine and semen samples from 181 men who were patients of an infertility clinic. These men had normal semen sperm concentration or slight oligozoospermia [71]. To measure the concentrations of PAHs present in the men, a PAH biomarker, 1-hydroxypyrene (1-OHP), was measured in the urine [71]. Sperm aneuploidy was measured in the semen samples [71]. Total sex chromosome disomy as well as chromosome-18 disomy has a positive association with level of 1-OHP in the urine [71]. 

Gaspari et al. [72] recruited 205 men through the Infertility Clinic of the University of Milan who had morphological abnormalities in the sperm and whose partners did not have known causes of infertility. The concentration of PAH–DNA adducts were measured in 182 of the men [72]. Higher levels of PAH–DNA adducts were observed in men with occupational exposure to PAHs [72]. Daily alcohol consumption had a negative association with PAH–DNA adducts (*p* = 0.01) [72]. Furthermore, PAH–DNA adducts had a positive association with morphological abnormalities of the head of the sperm (r = 0.30) [72]. In contrast, PAH–DNA adducts were negatively associated with abnormalities in the neck of the sperm (r = −0.21) [72]. Significantly more PAH–DNA adducts were observed in infertile men compared to fertile men (*p* = 0.04) suggesting that PAH–DNA damage plays a role in fertility [72]. 

### 7.2. Female-Specific Responses

Windham et al. [73] collected urine samples daily during menstrual cycles of heavy smokers (20 cigarettes per day) and non-smokers and measured progesterone and estrogen. Women categorized as heavy smokers averaged a cycle 2.6 day shorter mainly attributed to the shortened follicular phase [73]. Ex-smokers who smoked for ten or more years had shorter luteal phases than women who had never smoked [73]. Based on the findings of the altered length of phases in the menstrual cycle, Windham et al. [73] suggest that heavy smoking can play a role in subfecundity and early menopause.

Neal et al. [74] compared the levels of BaP as well as other PAHs found in cigarette smoke in the serum and follicular fluid of 19 women exposed to mainstream smoke (smoker), 7 exposed to side stream smoke (partner smokes), and 10 non-smokers. Significantly higher levels of BaP was found in the follicular fluid of women exposed to mainstream smoke (1.32 ± 0.68 ng/mL) in comparison to the two other groups (side stream exposed (0.05 ± 0.01 ng/mL) and non-smoking (0.03 ± 0.01 ng/mL)) [74]. Moreover, in the mainstream smoke group levels of BaP were positively correlated with the number of cigarettes reportedly smoked [74]. Interestingly, Neal et al. [74] found that levels of BaP in the follicular fluid were significantly higher (*p* < 0.001) in women who did not get pregnant through IVF versus the women who did [74]. Seyler et al. [75] found no change in levels of secretion of LH or FSH after patients smoking two high nicotine cigarettes.

Archibong et al. [76] exposed female rats to 50, 75, or 100 μg BaP/m^3^ via inhalation for 4 h a day for 14 days. After exposure to 100 μg BaP/m_3_ number of pups per litter as well as ovulation rate significantly decreased (*p* < 0.002) [76]. Furthermore, the pro-estrous stage of the estrous cycle was lengthened by approximately 24 h compared to the other exposure groups (*p* < 0.05) [76]. Plasma concentrations of estradiol significantly decreased in all exposed groups compared to controls (*p* < 0.05) [76]. Progesterone levels were only significantly decreased in the BaP exposed groups during diestrus 1 in comparison to controls (*p* < 0.01) [76]. During pro-estrous in exposed groups, circulating LH and estradiol concentrations were reduced [76]. In contrast, plasma FSH concentrations were increased in exposed groups during diestrus I, diestrus, II, estrus, and proestrus [76]. 

Luderer et al. [60] recruited 51 women between the ages of 18 and 44 years living in Orange County, California. They collected daily urine samples from all participants in order to measure urinary LH and estrone 3-glucuronide (E_1_3G). On cycle day 10 urine samples were collected to measure nine metabolites of PAH, categorized as hydroxylated PAHs (OH-PAH) [60]. Furthermore, the length of the follicular phase was positively associated with two of the OH-PAHs measured, and follicular LH concentrations had a negative association with other OH-PAHs analyzed [60].

Human subcellular ovary samples (*n* = 8) were incubated with two doses of BaP, 1 μM or 3 μM, in order to measure BaP ovarian toxicity [67]. Ovary samples were obtained from women who underwent operations to remove uterine tumors [67]. The tissue incubated with 3 µM created significantly more BaP metabolites than the tissue incubated with 1 µM [67]. With the small sample size and fact that samples come from women suffering with uterine tumors taken into consideration, Rekhadevi et al. [67] concludes that the body can metabolize BaP, and the metabolites are able to accumulate in reproductive organs. 

## 8. Cadmium and Lead

Cadmium (Cd) exposure in the environment comes from forest fires, waste incineration, weathering consumption of rocks, sea aerosols, mobilization from soils and landfills, batteries, pigments, plastic stabilizers, pesticides, fertilizers, fossil combustion, and volcanic activity [77]. Depending on the proximity to an industrial site, the range of concentrations of Cd in the air vary [77]. At the site of emission, the concentration can be found up to 100 ng/m^3^ while in remote areas up to 0.1 ng/m^3^ can be found in the air [77]. Cd enters the food chain through bioaccumulation in organic matter. The IARC estimates that the average daily intake of Cd from food is between 0.1–0.4 ug/kg body weight [78]. In the general population, blood plasma Cd (bpCd) concentration is within the range of 0.4–1 ug/L in non-smokers and 1.4–4 ug/L in smokers; nevertheless, higher concentrations have been reported in environmentally contaminated areas (>10 ug/L) [78]. Occupational exposure to Cd consists of inhalation of Cd-polluted fumes or dust and ingestion through dust-contaminated hands [77]. Cd can bind to estrogen receptors and androgen receptors and cause strong estrogenic and androgenic responses [79]. 

Lead (Pb) pollution in the air can be due to smelter plants that emit Pb dust in the air to then settle in the soil and leaded gas which creates fine leaded dust emitted from automobile exhaust pipes [80]. Leaded gasoline is responsible for remnants of lead to account for 4 to 5 million metric tons of lead in the environment [81,82]. Human exposure to Pb and Cd can occur through inhalation of fossil fuel combustion products, drinking water contaminated by Pb used in pipes, and ingestion of flakes of Pb-based paints, and once the body absorbs these metals, they collect in bones, kidneys, and reproductive organs [83]. Prenatal and postnatal exposure can also occur through the placenta and milk, respectively [84]. Interestingly, in 2000 maternal–infant pairs comparing partitions of lead in cord blood, cord tissue, placenta, and milk, Needham et al. [84] found that the placenta had the highest lead concentrations. Exposure to Pb and Cd occurs through contaminated water, food, and air, and the amounts of Pb and Cd in the environment have increased due to more use of gasoline and petroleum, industrialization, and smoking [83]. Pb and Cd negatively affect fertility by altering the HPG axis or by damaging testicular tissues directly [78,85]. By altering the normal function of the HPG axis, Pb and Cd disrupt spermatogenesis, spermiogenesis, and steroidogenesis [85,86]. The studies described below are compiled and presented in Table 1 and Table 2.

### 8.1. Male-Specific Responses

Wijesekara et al. [83] studied the effects of environmental exposure versus occupational exposure to lead (Pb) and cadmium (Cd). Environmental exposure was defined as living less than 50 m from a main road or industrial site emitting toxicants; men working as welders, painters, printers, or farmers using agrochemicals were described as occupationally exposed [83]. Of the total population, mean Pb concentrations in seminal plasma was 15.77 ug/dl and Cd concentration was 1.18 ug/dl. The highest mean Pb concentration (19.7 µg/dl) (*p* = 0.1) was observed in the environmentally exposed group, while the highest mean concentration of Cd (1.4 µg/dl) (*p* = 0.3) was observed in the occupationally exposed group [83]. Normal sperm motility was associated with concentrations of Pb = 15.04 ug/dl (not significant) and Cd = 1.15 ug/dl (not significant), while sperm with reduced motility was associated with concentrations of Pb = 17.25 ug/dl and Cd = 1.25 ug/dl [83].

Telisman et al. [87] found in 149 men between the ages of 20 and 43 with occupational exposure to Pb that there was a significant (*p* < 0.05) decrease in sperm density, motility and viability. Increased levels of serum testosterone and estradiol were also found in these men [87]. Similarly, blood concentrations of Cd were associated with decreased sperm motility and increased abnormal sperm morphology and serum testosterone (*p* < 0.5) [87]. Neither Pb nor Cd influenced concentrations of FSH or LH in blood [87]. Moderate exposure to Pb (blood Pb < 400 mg/L) and Cd (blood Cd < 10 mg/L) can cause a decrease in human semen quality [87].

### 8.2. Female-Specific Responses

Proestrus rats were exposed intraperitoneally with 0.05 mg/kg/day with Pb acetate, Cd acetate, or in combination for 15 days [88]. In Cd and combined treatments, LH and FSH levels were decreased compared to controls, while Pb exposure alone showed no significant changes [88]. After treatment an increased accumulation of Pb and Cd was seen in the hypothalamus and pituitary [88], and the pituitary membrane fluidity decreased [89]. Specifically, Pillai et al. [89] showed that in rats the fluidity of the pituitary membrane decreased after Pb exposure to 0.05 mg/kg/day. These findings suggest that the regulatory mechanisms of the hypothalamic-pituitary axis are affected by metal accumulation whereas combined metal treatments are not additive [88].

Cheng et al. [90] administered CdCl_2_ by injection to 6- to 8-week-old female mice. For seven consecutive days, treatment group mice received doses of 0.5, 1, 1.5, 2, 3, or 5 mg Cd/kg body mass [90]. The increase in dose had a negative association with fertilization rate [90]. In vivo fertilized zygotes were analyzed and cultured in order to reach the blastocyst stage [90]. In the treatment group, 51.85% reached the blastocyst stage, while 82.61% did in the control group, showing oocyte maturation was inhibited by Cd [90]. Furthermore, treatment group 3 mg Cd/kg showed significantly reduced fecundity when caged with male mice in comparison to controls [90]. 

Tanrikut et al. [91] examined endometrial samples in thirty-three women who had unexplained infertility and compared those samples to samples from thirty-two fertile women. In thirty of the thirty-three women with infertility, Cd was detected in endometrial tissue in comparison to just eleven of thirty-two women who were fertile [91]. Moreover, the interquartile range of endometrial Cd was 1.46–30.23 µg/L versus 0.00–0.40 µg/L in women with infertility and fertile women, respectively [91]. Similarly, in five of the thirty-three women who had unexplained fertility, Pb was observed, while only one of the thirty-two women who were fertile had detectable Pb in the endometrial sample [91]. 

Gollenberg et al. [92] analyzed blood and urine samples from 705 peripubertal girls ages 6–11 years for associations between inhibin B, a marker for follicular development, and LH and Cd and Pb. Inhibin B levels were negatively associated with Pb levels [92]. Data showed that girls with high Pb and high Cd levels had lower inhibin B concentrations in comparison to girls with only high Pb or girls with low Pb and low Cd [92]. 

## 9. TCDD

2,3,7,8-tetrachlorodibenzo-p-dioxin (TCDD) is an anthropogenic halogenated dioxin, which is an unintended by-product from burning processes and phenoxy herbicide production [93]. An embryo is more vulnerable to small doses during development than its mother; exposure in utero and through lactation can cause developmental problems without the mother being affected [93,94]. During development, TCDD does not appear to have estrogenic or anti-estrogenic action, rather it acts on specific tissues in the HPG axis [93]. Exposure to TCDD during the perinatal period can affect structural and functional development of reproductive organs by interfering with hormone-mediated events [94]. Although TCDD control was implemented at the end of the 1970s due to its long half-life and ability to biomagnify in the food web, animal exposure still occurs [95]. The studies described below are compiled and presented in Table 1 and Table 2.

### 9.1. Male-Specific Responses

On gestational day (GD) 15, pregnant rats were treated with 0.7 µg/kg TCDD via gavage [94]. On postnatal day 21, offspring were weaned [94]. Plasma testosterone and LH concentrations did not significantly differ between controls and the exposed groups [94].

Lin et al. [96] examined the effects of TCDD on aryl hydrocarbon receptor (AhR) knockout (KO) mice versus wild-type mice. On GD 13, pregnant mice were administered a single oral dose of 5 µg/kg TCDD [96]. Pups remained with their mothers until weaning on PND 21 [96]. Exposure to TCDD in utero and during lactation did not have an effect on sperm production on AhRKO or wild type [96]. However, wild-type mice exposed in utero and during lactation had a reduced absolute testicular weight on PND 35 compared to AhRKO mice [96]. The results suggest that TCDD caused AhR independent and dependent effects [96]. Fukuzawa et al. [97] found in male mice treated with 0, 0.8, 4, 20, or 100 μg/kg TCDD bw after exposure to TCDD, p45-scc and luteinizing hormone receptors (LHR) levels in the testis were reduced in a dose-dependent comparison to controls that were AhR null, suggesting that the mechanism of action involves aryl hydrocarbon receptor. 

Boda et al. [98] found a high correlation between 2,3,7,8-TCDD concentrations in cord blood and breast milk. This finding suggests that the levels found in breast milk represent the levels of TCDD exposure prenatally [98]. A reduction in testosterone in cord blood was seen in >/= 5.5 pg/g lipid 2,3,7,8-TCDD exposure in boys prenatally [98].

Twenty-two years after the explosion in Seveso, Italy, that left the near population exposed to TCDD, Mocarelli et al. [99] examined sperm and serum samples in 397 men who were exposed to TCDD during infancy/prepuberty (1–9 years of age), puberty (10–17 years of age), or adulthood (18–26 years of age). Healthy volunteers consisted of 372 men who were the same age as the exposed individuals, but they did not live in the TCDD-contaminated areas during the explosion in 1976 [99]. In the infancy/prepuberty groups exposed to TCDD, sperm count (*p* = 0.025), progressive sperm motility (*p* < 0.001), and total number of motile (*p* = 0.018) sperm were significantly decreased compared to the control group [99]. In contrast, in the group exposed during adulthood, no significant differences were found in the sperm variables compared to the controls [99]. 

Mocarelli et al. [99] also examined hormone levels and found that in the infant/prepuberty and puberty group serum, 17β-estradiol (E_2_) concentrations were lower than in controls, while serum FSH concentrations were higher than in the controls. In contrast, the adulthood group had no significantly different hormonal levels than in controls [99]. The findings of this study suggest that infantile and prepubescent males are more susceptible to negative effects in sperm and hormone concentrations due to TCDD exposure the pubescent and adult males [99]. Furthermore, low concentrations of TCDD affect the endocrine system and leads to a reduction in E_2_ affecting semen quality [99]. Infancy and prepuberty males are more vulnerable to exposure than males during puberty and adulthood [99]. 

### 9.2. Female-Specific Responses

The role of aryl hydrocarbon receptors in female reproduction is to regulate the expression (by activating transcription) of ovarian P450 aromatase (Cyp19), a key enzyme in estrogen synthesis, in granulosa cells [100]. Baba and colleagues [100] found that the AhR ligand 9,10-dimethyl-1,2-benxanthracene (DMBA) induced Cyp19 expression in female mice causing increased levels of estradiol. Baba and colleagues [100] propose that disruptions in the AhR ligand is the endocrine-disrupting mechanism of action of other dioxins. Meanwhile, Moran et al. [101] found that TCDD targets p450c17, suppressing its expression, which inhibits the secretion of E2 from human luteinized granulosa cells. Moreover, Baldridge et al. [102] found that TCDD effects on E2 secretion were time dependent, not dose dependent, and the decreased secretion of E2 in human luteinizing granulosa cells was a result of decreased expression of CYP11A1 and CYP19A1. 

Endometriosis is defined as stromal and/or endometrial glandular epithelium implants outside the uterine cavity with symptoms including pelvic pain, dysmenorrhea, dyspareunia, dyschezia, urinary symptoms and even asymptomatic [103]. Two hypotheses for the pathogenesis of the disease involve the retrograde menstruation. High concentrations of AhR can be found in the endometrium and in immune cells [103]. Normally endometrial tissue is cleared out of the peritoneal cavity by immune system cells; however, dioxin-like environmental chemicals can alter the inflammatory processes responsible for clearing the tissue out, which could cause the development and build up of endometrial tissue [103]. The material metalloproteinase (MMP) system degrades extracellular matrices, and its alterations in its expression has been studied in endometriosis [104]. In healthy tissue, progesterone downregulates the expression of MMPs [103]. TCDD exposure to endometrial tissue results in the production of matrix metalloproteinases (MMPs) [103] as well as decreases the amount of progesterone leading to an increased expression of MMPs in the endometrium [105]. 

Mice antral follicles were exposed to TCDD in doses of 0.1, 1, 10, and 100 nM for 96 h in a project examining the ovarian toxicant by [106]. The secretion of progesterone (PR), androstenedione (A4), testosterone (T), and 17β-estradiol (E2) into the media was analyzed after the 96 h [106]. Exposure dose of 1nM was the only dose tested that all four hormones decreased in concert [106]. PR showed the greatest decrease at a dose of 1 nM [106]. A significant decrease in secretion was seen in A4 and T at the lowest doses 0.1 and 1 nM [106]. After exposure to 0.1, 1, and 10 nM E2 secretion levels decreased [106]. A significant increase in Cyp1b1, an enzyme responsible in AHR pathway activation, was seen in antral follicles after 96 h of culture regardless of the dose [106]. Furthermore, the expression level of Ahr mRNA was not affected by TCDD by any dose tested [106]. 

Pesonen et al. [107] administered 0.04, 0.2, or 1.0 µg/kg TCDD via gavage to pregnant rats on GD 13. On PND 14 offspring were sacrificed and ovaries were collected and cultured for observation [107]. No significant differences in FSH or E2 levels were observed between TCDD treatment groups and controls [107]. After 5 days in culture, E2 levels were decreased in exposure group 1.0 µg TCDD/kg [107]. In an earlier study, maternal TCDD exposure inhibited ovarian P450arom activity and P450arom, P450scc, and StAR mRNA levels which could explain the decrease in E2 levels [108].

On GD 15.5, pregnant mice were treated with 10 µg/kg TCDD via gavage [109]. In these mice the half-life of TCDD is approximately 11 days [110]; therefore, offspring were exposed in utero and during lactation [109]. Double and triple exposed mice received another treatment of 10 µg/kg TCDD at 4 weeks of age or at 4 weeks and 9 weeks, respectively [109]. No mice treated three times achieved pregnancy [109]. Progesterone (PR) A and B proteins were measured in the uterus, and in F1 mice who were infertile after exposure in utero had decreased expression of PR [109]. Likewise, F1 mice exposed in utero and prior to puberty had a decrease in expression of PR and fertility [109]. The decrease in PR expression appeared to negatively affect fertility [109].

King Heiden et al. [111] fed zebrafish 0, 10, 40, or 100 ng of TCDD/g food (ppb) for 5 days a week for 4 weeks. 6 females from each exposure group were sacrificed after 5, 10, 15, and 20 days of exposure [111]. Females exposed to 40 and 100 ppb TCDD had a significant decrease in egg production and spawning success (*p* = 0.04 and <0.0001, respectively) [111]. After 5 days of exposure, the 40 and 100 ppb exposure group had significantly decreased levels of serum E2 (>36% decrease compared to controls), and after 10 and 15 days of exposure all of the treatment groups showed a significant decrease in serum E2 (>50% decrease) [111]. After 20 days of exposure to 100 ppb TCDD the total number of follicles was significantly decreased (84% reduction) [111]. Furthermore, the 40 and 100 ppm exposure group had significantly smaller secondary growth follicles in comparison to the controls (8–10% decrease) [111]. An increase in follicular atresia was observed in a dose-dependent manner [111]. This study demonstrates that TCDD exposure impairs follicular development and increases the number of atretic follicles suggesting ovarian toxicity [111].

## 10. Polychlorinated Biphenyls

Polychlorinated biphenyls (PCBs) are persistent organic pollutants [112] formerly used as coolants, plasticizers in paint and cements, and flame retardants [113,114] which contaminate food, water, and air [115]. Production of PCBs stopped worldwide in the late 1970s to early 1980s due to their persistence, ability to bioaccumulate, and toxicity [116]. Based on the placement and number of chlorines, 209 congeners exist, when there is no Cl in the ortho position, “dioxin-like” toxicity can occur [116]; thus, these congeners are referred to as dioxin like [114]. Interestingly, increased serum concentrations of PCBs in individuals often indicate that the individual has higher than average concentrations of other POPs [117]. Mori et al. [118] found that infants younger than 2 years old who were breastfed had levels of PCBs similar to individuals over 60 years old. Asante et al. [38] found in breast milk samples collected from 2004 to 2009 that the mean concentration of PCBs was 62 ng/g lipid weight (lw) while the range was 15–160 ng/g lw. We included human studies examining the effects of PCB exposure. It is not specified in these studies how the patients were exposed; nonetheless, these studies show the effects of PCB exposures. The studies described below are compiled and presented in Table 1 and Table 2.

### 10.1. Male-Specific Responses

Burns et al. [119] enrolled 473 Russian boys of 8–9 years old and measured the serum levels of dioxin-like compounds and PCBs. The subjects were assessed annually for stage of puberty (until age 17–18); 315 of the 473 completed the study [119]. PCB levels in serum and timing of puberty suggested that exposure to non-dioxin-like PCB congeners advanced the timing of male puberty [119].

Molto et al. [120] sampled the blood serum of 21 men visiting a fertility clinic suffering from different reproductive problems. The results showed that dioxin-like (DL) PCB congeners 118, 156, and 105 were responsible for 67% of the mean concentration of total DL-PCBs in serum samples in male patients [120]. Molto et al. [120] also found that some PCB levels in serum are positively correlated to fat mass while others are not. These findings were attributed to Ronn et al. [121], who found that low-chlorinated PCBs were positively correlated to fat mass, while highly chlorinated compounds were negatively correlated. This study was the first of its kind, and therefore these were the first data collected as of 2015, and the data found in infertile serum samples of infertile men were not compared to serum samples of fertile men [120].

Peterson et al. [122] performed a physical examination and collected a blood and semen sample as well as a questionnaire from 241 Faroese men born between January 1981 and December 1984; an additional 21 Faroese men participated providing everything but a semen sample. A positive association was found between PCB concentrations in serum and sex hormone-binding globulin (SHBG) and LH [122]. Furthermore, the total testosterone to total estradiol ratio was higher when the levels of PCBs were higher (*p* = 0.01) (Peterson et al., 2018). These results suggest that the higher levels of LH and total T are due to altered levels of SHBG [122]. No significant association was found between PCB concentrations and semen parameters [122].

Ferguson et al. [123] examined 341 men from a US infertility clinic for associations between PBCs, hexachlorobenzene (HCB), and p,p′-DDE and serum sex hormone levels. PCB-118 as well as dioxin-like PCBs had a negative association with SHBG and total testosterone [123]. Likewise, McAuliffe et al. [124] examined concentrations of 57 PCB congeners and dichlorodiphenyldichloroethylene (p,p′-DDE) in 192 men from subfertile couples. Chromosomes X, Y, and 18 were analyzed with fluorescence in situ hybridization to determine XX, YY, XY, and total sex chromosome disomy in sperm nuclei [124]. A positive correlation was found between incidence rate ratios (IRRs) for XX, XY, and total sex chromosome disomy and increasing quartiles of p,p′-DDE [124]. Furthermore, a positive correlation was also observed between IRRs for XY and total sex chromosome disomy and increasing quartiles of PCBs [124]. 

### 10.2. Female-Specific Responses

On embryonic days 16 and 18 (critical period of neuroendocrine development), pregnant Sprague–Dawley rats were treated with 0, 0.1, 1, or 10 mg/kg PCB mixture, Aroclor (A) 1221 [125]. The only significant alteration seen in F1 females came from the 1 mg/kg A1221 group where LH serum levels were significantly increased (*p* < 0.05) [125]. No significant differences were seen in serum estradiol or progesterone or uterine and ovarian weights [125]. 

In an endometriosis mouse model experiment, mice were treated with PCB126 (0.03 or 0.3 mg/kg) or PCB153 (50 mg/kg via gavage) two days before the endometrium injection which induced endometriosis [126]. Exposure to PCB126 resulted in a significant increase in endometriotic lesions at 10 days post-exposure; however, at 20 days post-exposure lesions had decreased to levels equivalent to the control group [126].

In human endometriosis cultured cells, differences in E2 levels and HSD17B7 mRNA expression depended on whether they were exposed to dioxin-like (PCB126) or non-dioxin-like (PCB153) PCB congeners [126]. E2 levels increased with exposure to PCB126 [126]. After studying the same congeners in endometriosis mouse models, Huang et al. [126] found that dioxin-like PCB (PCB153) exposure was associated with endometriotic lesions at 10 days post-exposure while non-dioxin-like PCB exposure did not have an association.

## 11. Discussion

Herein, we have compiled studies describing the effects of endocrine-disrupting compounds for which human exposure through inhalation is plausible. These studies show that anthropogenic pollutants affect the HPG axis and/or subsequent fertility. These effects are most prominent in studies of EDCs in mouse models. Inhalation was not the exposure route in all the studies; however, all of the studies do represent the interaction of the EDC with specific receptors, hormones, or other means of endocrine disruption. Thus, while the dose leading to endocrine disruption will vary according to exposure route, all the studies presented herein still represent a mechanism of action on the HPG axis and/or subsequent consequence to fertility.

Flame retardants have effects in rodent and human models. In men, higher concentrations of BDE209 were associated with lower testosterone levels and lower sperm concentration and motility [29]. Semen parameters were affected by BDE47, BDE 100, and BDE 153 [32]. Meeker et al. [30] found that BDE 47, 99, and 100 were inversely related to FSH and LH serum concentrations. In rats, BDE exposures during gestation did not affect male offspring testosterone or LH [34]. 

In humans, concentration of BDE congeners did not have an effect on menstrual cycle characteristics. However, they did affect the time needed to get pregnant [42]. The PFR metabolite BDCIPP showed a negative correlation with the number of best-quality embryos [40]. Likewise, in women undergoing IVF, PFR metabolite concentrations and success of IVF were directly correlated. In female rats, Lefevre et al. [37] showed that BFR exposure through the diet during gestation can increase testosterone levels and alter folliculogenesis. Talsness et al. [41] showed that in utero and lactational exposure to high doses of PBDE-47 alter folliculogenesis and serum estradiol levels in female rats without affecting reproduction. 

Rodent studies indicate that diesel exhaust particle exposure is associated with myriad effects. In male rats, DEP exposure in utero is linked to alterations in gene expression that affect sperm production [44]. This exposure can also alter FSH serum levels, testosterone, and estradiol levels [51]. Acute DEP exposure in murine Leydig TM3 cells may result in a decrease in ERα mRNA expression [55]. In female mice, Tsukue et al. [57] found that DEP exposure affects uterine, thymus, and ovary weights. Mouse exposure perinatally and postnatally to the daily average PM_2.5_ levels humans are exposed to as reported by the World Health Organization (WHO) resulted in a reduction in primordial follicles [58]. 

PAH exposure is ubiquitous and associated with multiple effects on the HPG axis; however, concentrations of PAH metabolites in men differed depending on rural or industrial city exposure [66]. The metabolite 1-OP showed a positive correlation with serum LH levels [70]; 1-OHP was positively associated with chromosome-18 disomy and total sex chromosome disomy [71]. Number of PAH–DNA adducts and morphological abnormalities in the head of a sperm were positively correlated with occupational PAH exposure in a study by [72]. Women who smoke or who are ex-smokers had altered lengths of the follicular phase and luteal phase, respectively, in comparison to women who had never smoked [73]. In rats, after exposure to BaP, the pro-estrous stage was lengthened, and LH and estradiol concentrations were altered [76]. Luderer et al. [60] found that certain PAH metabolites had a positive association with the length of the follicular phase. Furthermore, Neal et al. [74] found significantly higher levels of BaP in the follicular fluid in women who smoke in comparison to women exposed to second-hand smoke and non-smokers. Ovarian subcellular tissue taken from women with uterine tumors showed that BaP metabolites can accumulate in the ovaries [67]. 

Cadmium and lead are two of metals for which inhalation is a significant exposure route. Other metals such as mercury may also effect fertility in women [127]; however, not as much research has focused on this metal. In humans, reduced sperm motility was associated with concentrations of Cd and Pb lower than the highest concentrations found in men with occupational and environmental exposure, respectively [83]. Telisman et al. [87] also found in occupationally exposed men, Pb was associated with a decrease in sperm density and motility as well as viable sperm. Moreover, no significant association was observed between levels of Pb or Cd and concentrations of FSH or LH; however, an increase in serum testosterone and estradiol was observed in these men [87]. Benvenga et al. [128] found that myoinositol and seleno-methionine can have protective effects on testicular damage caused by Cd. Combined exposure to Pb and Cd can decrease the levels of LH and FSH in proestrus rats [88], and Pb treatments alone can decrease the fluidity of the pituitary membrane [89]. In mice, increased doses of Cd were associated with infertility [90]. In human endometrial samples, Cd and Pb concentrations appeared to have a relationship with infertility [91]. In urine of peripubertal girls, negative associations were found when measuring concentrations of Pb and Cd together and inhibin B [92]. 

Inhalation of TCDD has the potential to affect fertility through the HPG axis. In male mouse models, Lin et al. [96] studied the effects of TCDD exposure at relatively high doses and found that in comparison to AhRKO wild-type mice in utero and lactational exposure led to reduced absolute testicular weight. To explore the role of the AhR in TCDD exposure, Fukuzawa et al. [97] examined the testis and found LHR levels decreased in male mice in comparison to AhR-null mice. In human males, Boda et al. [98] found that exposure to TCDD can occur through breast milk. Adulthood exposure to TCDD appears to have no significant effect on sex hormone levels or sperm variables such as sperm count, progressive sperm motility, and total number of motile sperm while infancy and prepubertal exposure does [99]. Cultured antral follicles exposed to TCDD secrete fewer amounts of various sex hormones [106]. In utero exposure to TCDD affects the ovarian secretion of E2 in rats [107], and in mice in utero exposure affected PR expression [109]. In zebrafish, TCDD exposure affected egg production, levels of serum E2, and number of follicles [111].

Exposures to various PCB congeners can affect hormone levels, puberty, and even chromosome disomy [119,120,122,124]. Exposure to non-dioxin-like PCBs may advance the timing of male puberty [119], and in infertile males, PCB congeners 118, 156, and 105 were found to contribute to over half of the total concentration of dioxin-like PCBs in serum [120]. PCB concentrations in serum appear to be positively correlated with SHBG, which may cause an elevation in total T and LH without affecting sperm quality [122]. Furthermore, higher levels of PCBs may affect sex chromosome disomy [124]. 

## 12. Conclusions

Countries around the world have banned many EDCs or restricted use and release to the environment; however, air, soil and water still contain compounds that affect the HPG axis and subsequent fertility. Humans are potentially exposed to multiple EDCs through inhalation. Herein, we have summarized the effects of prominent individual EDCs for which inhalation is a plausible pathway. All compounds described herein indicate potential for endocrine disruption via inhalation. However, few studies have studied the effects that mixed EDC exposure has on the HPG axis and fertility. Conducting mixture toxicity studies is inherently difficult to conduct and extrapolate to humans. Thus, the lack of mixture toxicity studies is glaring and an obvious data gap in the understanding of the effects of EDCs on the HPG axis and fertility. While the literature is replete with indications that multiple, inhalable toxicants act as EDCs, studies on mixtures of EDCs is imperative to gain a more accurate picture of how EDC exposure affects the HPG axis and fertility. The studies described below are compiled and presented in Table 1 and Table 2.

**Table 1:** Summary of Inhalable Endocrine-Disrupting Chemicals and Female-Specific Effects in Multiple Animal Models.

Chemical Measured	Model	Examples/Exposure	Effect *	Citation
BDE 209	human	samples taken from men at fertility clinics	inversely related to testosterone levels	[29]
			higher levels associated with greater risk of subfertility	
BDE 28			inversely related to testosterone levels	
BDE153			positive correlation with testosterone and estradiol	[29]
			negative correlation with sperm concentrations and testis size	[32]
BDE 47, 99, 100			negative correlation with FSH and LH serum	[30]
BDE154		prenatal exposure	testosterone levels positively associated with serum concentrations of BDE154 in mother’s serum	[39]
BDCIPP		male from couple undergoing IVF	concentrations correlated with decrease in number of best-quality embryos	[40]
BDE99	rat	GD6, doses: 0.06 or 0.3 mg BDE99/kg	no effect	[34]
BaP		inhalation	decreased concentrations of plasma testosterone	[69]
		24 and 48 h exposure to 75 ug BaP/m^3^		
		24,48, and 72 h exposure to 75 ug BaP/m^3^	increased LH concentration	
1-OP	human	urine from infertile men	positive correlation between serum LH and urine concentrations of 1-OP (*p* = 0.048)	[70]
TI-1 and TII-2		semen samples	inverse relationship between sperm motility (confidence interval = 95%)	[66]
1-OHP		urine and semen samples from patients of infertility clinic	positive association between levels of 1-OHP in urine and total sex chromosome disomy and chromosome-18 disomy	[71]
PAH–DNA adducts		men from infertility clinic	positive associations with abnormalities of head of sperm (r = 0.30)	[72]
			negative associations with abnormalities in neck of the sperm (r = −0.21)	
PCBs		serum from boys over time	advances time of puberty	[119]
		serum from Faroese men	total testosterone and total estradiol ratio increase (*p* = 0.01)	[122]
PCB-118		serum from men in US infertility clinic	testosterone levels positively associated with serum concentrations of BDE154 in mother’s serum	[123]
p,p′-DDE		subfertile couples	positive correlation with total sex chromosome disomy in sperm nuclei	[124]
			positive correlation with IRRs	
DE	rats	inhalation of DE	decrease in LH (*p* < 0.05)	[51]
			increase in testosterone and estradiol (*p* < 0.05)	
	cells	inhalation of high NR-DE	increases testosterone	[54]
		F-DE	decreased testosterone	
DEP		murine Leydig TMC cells	decrease in ERα mRNA expression (*p* < 0.01)	[55]
			increase in P450 1A1 mRNA expression (*p* < 0.01)	
Cd	human	occupational exposure	blood Cd associated with decreased serum testosterone (*p* < 0.05)	[87]
TCDD		exposed during infancy and prepuberty	decreased sperm count (*p* = 0.025)	[99]
			decreased sperm motility (*p* < 0.001)	
			decreased number of total number of motile sperm (*p* = 0.018)	

* *p*-values listed depending on the details given in each study.

**Table 2:** Summary of Inhalable Endocrine-Disrupting Chemicals and Female-Specific Effects in Multiple Animal Models.

Chemical Measured	Model	Matrix/Exposure	Effect *	Citation
BFR	rat	diet 2–3 weeks before mating until GD20	increase in preantral and antral follicles	[37]
		dose: 0.06, 20, or 60 mg/kg/day	enlargements of antral follicles	
PBDE-47		drinking water from GD6-PND21	reduction in ovarian weight	[41]
		dose: 140 ug/kg bw		
		700 ug/kg bw	decrease in tertiary follicles and serum estradiol concentrations	
BDE 47, 99, 100, and 153	human	blood	no relationship between congener presence and menstrual cycle	[42]
			positive association between congener levels and time till pregnant	
BDCIPP, DHPH, ip-PPP, tb-PPP, BCIPP		urine from women undergoing IVF	negative association between sum of metabolites and successful IVF	[43]
n/a		urine from heavy smokers	heavy smokers had shorter follicular phase	[73]
BaP		serum and follicular fluid	higher levels led to unsuccessful IVF (*p* < 0.001)	[74]
OH-PAH		urine	negatively associated with follicular LH concentrations	[60]
BaP	rats	inhalation	decreased progesterone (*p* < 0.01), LH, and estradiol levels	[76]
		50, 75, 100 μg BaP/m^3^		
			increased FSH concentration	
		100 μg BaP/m^3^	decreased number of pups per litter (*p* < 0.002)	
			ovulation rate decreased	
			lengthened pro-estrous cycle (*p* < 0.05)	
PCB		embryonic days 16 and 18	increased LH levels (*p* < 0.05)	[125]
		pregnant rat treated with 1 mg/kg		
PCB126	mouse	0.03 or 0.3 mg/kg	increase in endometriotic lesions 10 days post-exposure	[126]
DEP		3.0 mg DEP/m^3^	lower thymus and ovary weight	[57]
			earlier vaginal opening	
		1.0 mg DEP/m^3^	earlier vaginal opening	
DE		Pre- and postnatal	decrease in primary follicles	[58]
TCDD	zebrafish	40 and 100 ppb 5 days a week for 4 weeks	decrease in egg production (*p* = 0.04)	[111]
			8–10% decrease in size of secondary growth follicles	
			decrease in spawning success (*p* < 0.0001)	
		40 and 100 ppb 5 days	>36% decreased levels of serum E2	
		40 and 100 ppb 15 days	>50% decrease in serum E2	
		100 ppb 20 days	84% reduction in total number of follicles	

* *p*-values listed depending on the details given in each study.

## Abbreviations

AhR	aryl hydrocarbon receptor
AMH	anti-Mullerian hormone
Ar	androgen receptor
Atrazine	2-chloro-4-ethylamino-6-isopropulamino-1,3,5-triazine
BaP	benzo(a)pyrene
BDE	brominated diphenyl ethers
Cd	cadmium
DE	diesel exhaust
DEP	diesel exhaust particles
EDCs	endocrine-disrupting chemicals
EPA	Environmental Protection Agency
E2	17(beta) estradiol
FSH	follicle-stimulating hormone
fT	free testosterone
GD	gestational day
GnRH	gonadotropin release hormone
GnRHR	gonadotropin release hormone receptor
HPG	hypothalamus-pituitary-gonadal
LD	lactational day
LH	luteinizing hormone
PAH	polycyclic aromatic hydrocarbons
PCB	polychlorinated biphenyl
Pb	lead
PBDEs	polybrominated diphenyl ethers
PM	particulate matter
PND	postnatal day
POPs	persistent organic pollutants
Ppb	parts per billion
PR	progesterone
PRL	prolactin
SHBG	sex hormone-binding globulin
SVOCs	semi-volatile organic compounds
VOCs	volatile organic compounds
T	testosterone
TCDD	2,3,7,8-tetrachlorodibenzo-p-dioxin
